# Endothelial dysfunction in breast cancer survivors on aromatase inhibitors: changes over time

**DOI:** 10.1186/s40959-024-00227-z

**Published:** 2024-05-01

**Authors:** Adnan Shaaban, Ashley Petersen, Heather Beckwith, Natalia Florea, David A. Potter, Douglas Yee, Rachel I. Vogel, Daniel Duprez, Anne H. Blaes

**Affiliations:** 1 Department of Internal Medicine, Division of Hospital Medicine, The Ohio State University, OH Columbus , USA; 2 420 Delaware Street, S.E. MMC 480, Minneapolis, MN 55455 USA; 3 Division of Biostatistics and Health Data Science, University of Minnesota, Minneapolis, MN USA; 4 Department of Internal Medicine, Division of Hematology, Oncology and Transplantation, University of Minnesota, Minneapolis, MN USA; 5 Department on Internal Medicine, Division of Cardiology, University of Minnesota, Minneapolis, MN USA; 6 Department of Obstetrics, Gynecology and Women’s Health, Division of Gynecologic Oncology, University of Minnesota, Minneapolis, MN USA; 7 Department of Medicine, Division of Cardiology, University of Minnesota, Minneapolis, MN USA

**Keywords:** Cardiotoxicity, Breast cancer, Aromatase inhibitors, Endothelial function

## Abstract

**Background:**

Breast cancer is estimated to comprise about 290,560 new cases in 2022. Aromatase inhibitors (AIs) are recommended as adjuvant treatment for estrogen-receptor positive (ER+) breast carcinoma in postmenopausal women, which includes approximately two-thirds of all women with breast cancer. AIs inhibit the peripheral conversion of androgens to estrogen by deactivation of the aromatase enzyme, leading to a reduction in serum estrogen level in postmenopausal women with ER+ breast carcinoma. Estrogen is known for its cardiovascular (CV) protective properties through a variety of mechanisms including vasodilation of blood vessels and inhibition of vascular injury resulting in the prevention of atherosclerosis. In clinical trials and prospective cohorts, the long-term use of AIs can increase the risk for hypertension and hyperlipidemia. Studies demonstrate mixed results as to the impact of AIs on actual CV events and overall survival.

**Methods:**

A single arm longitudinal study of 14 postmenopausal women with ER+ breast cancer prescribed adjuvant AIs at the University of Minnesota (UMN). Subjects with a history of known tobacco use, hypertension, hyperlipidemia, and diabetes were excluded to eliminate potential confounding factors. Participants underwent routine labs, blood pressure assessments, and vascular testing at baseline (prior to starting AIs) and at six months. Vascular assessment was performed using the EndoPAT 2000 and HDI/PulseWave CR-2000 Cardiovascular Profiling System and pulse contour analysis on two occasions as previously described. Vascular measurements were conducted by one trained vascular technician. Assessments were performed in triplicate, and the mean indices were used for analyses. All subjects were on an AI at the follow-up visit. The protocol was approved by the UMN Institutional Review Board and all participants were provided written informed consent. Baseline and follow-up characteristics were compared using Wilcoxon signed-rank tests. Analyses were performed using R version 3.6.1 (R Foundation for Statistical Computing, Vienna, Austria).

**Results:**

After six months of AI treatment, EndoPAT® ratio declined to a median 1.12 (Q1: 0.85, Q3: 1.86; p = 0.045; Figure 1) and median estradiol levels decreased to 2 pg/mL (Q1: 2, Q3: 3; p=0.052). There was no evidence of association between change in EndoPAT® and change in estradiol level (p = 0.91). There were no statistically significant changes in small or large arterial elasticity.

**Conclusions:**

We hypothesize that long-term use of AI can lead to persistent endothelial dysfunction, and further investigation is necessary. In our study, patients were on AI for approximately 5-10 years. As a result, we do not have data on whether these changes, such as EndoPAT® ratio and the elasticity of small and large arterial, are reversible with discontinuation of AI. These findings set the stage for a larger study to more conclusively determine the association between AI exposure and cardiovascular outcomes. Further studies should evaluate for multivariate associations withmodifiable risk factors for CV disease.

## Background

Breast cancer is estimated to comprise about 290,560 new cases in 2022 [1]. Aromatase inhibitors (AIs) are recommended as adjuvant treatment for estrogen-receptor positive (ER+) breast carcinoma in postmenopausal women, which includes approximately two-thirds of all women with breast cancer [2].

AIs inhibit the peripheral conversion of androgens to estrogen by deactivation of the aromatase enzyme, leading to a reduction in serum estrogen level in postmenopausal women with ER + breast carcinoma [3–5]. Estrogen is known for its cardiovascular (CV) protective properties through a variety of mechanisms including vasodilation of blood vessels and inhibition of vascular injury resulting in the prevention of atherosclerosis [6]. In clinical trials and prospective cohorts, the long-term use of AIs can increase the risk for hypertension and hyperlipidemia. Studies demonstrate mixed results as to the impact of AIs on actual CV events and overall survival [7, 8].

We hypothesized that the use of AIs and the associated reduction in estrogen would result in endothelial dysfunction, a predictor of early CV disease in women with breast cancer. Endothelial dysfunction, identified by reactive hyperemia using Endo-PAT, a non-invasive device that measures arterial vasoreactivity by assessing the peripheral arterial tone (Zoll Itamar), has been associated with an increased risk of CV events, independent of the Framingham risk score [9]. With the rising number of pre- and postmenopausal women on AIs for five to ten years, understanding the long-term impact of AIs on blood vessels and CV risk in cancer survivors is vital.

## EndoPAT 2000

EndoPAT 2000, developed by Zoll Itamar, is a U.S. Food & Drug Administration approved non-invasive device that utilizes peripheral arterial tonometry (PAT) to measure pulse wave amplitude (PWA) abnormalities in response to hyperemia vasoreactivity [10, 11]. PWA abnormalities have been previously studied and found to be associated with endothelial dysfunction and CV disease [10, 12, 13].

## Methods

We conducted a single arm longitudinal study of 14 postmenopausal women with ER + breast cancer prescribed adjuvant AI at the University of Minnesota (UMN). Subjects with a history of known tobacco use, hypertension, hyperlipidemia, and diabetes were excluded to eliminate potential confounding factors. Participants underwent routine labs, blood pressure assessments, and vascular testing at baseline (prior to starting AIs) and at six months. Vascular assessment was performed using the EndoPAT 2000 and HDI/PulseWave CR-2000 Cardiovascular Profiling System (Hypertension Diagnostic Inc., Eagan, MN) and pulse contour analysis on two occasions as previously described [14]. Vascular measurements were conducted by one trained vascular technician (NF). Assessments were performed in triplicate, and the mean indices were used for analyses. Biomarkers were obtained using a fasting blood draw to evaluate lipids, total cholesterol (TC), low-density lipoprotein cholesterol (LDL), high-density lipoprotein cholesterol (HDL), triglycerides (TG), high sensitivity CRP (hsCRP), serum glucose, 17-betaestradiol (estradiol), von Willebrand factor, tissue plasminogen activator, and plasminogen activator inhibitor-1. All subjects were on an AI at the follow-up visit. The protocol was approved by the UMN Institutional Review Board and all participants were provided written informed consent. Baseline and follow-up characteristics were compared using Wilcoxon signed-rank tests. Analyses were performed using R version 3.6.1 (R Foundation for Statistical Computing, Vienna, Austria).

## Results

Nine (64.3%) of the 14 participants had stage I breast cancer, four (28.6%) had stage II disease, and one (7.1%) had stage III disease. All fourteen received neoadjuvant or adjuvant chemotherapy. Ten (71.4%) participants received radiation therapy (four left-sided, six right-sided). Anastrozole was the most commonly used AI (6; 42.9%) followed by letrozole (5; 35.7%) and exemestane (3; 21.4%). None of the participants were on tamoxifen.

Patient characteristics and outcomes are outlined in Table 1. All patients were postmenopausal women. A total of 20 patients met inclusion criteria; however, only 14 patients who had follow-up visits were included in the analyses. Median baseline age of participants was 58 years (1st quartile [Q1]: 56, 3rd quartile [Q3]: 60) and median baseline body mass index was 26.5 kg/m^2^ (Q1: 24.4, Q3: 31.6). Median systolic and diastolic blood pressure was 120 (Q1: 115, Q3: 124) and 70 (Q1: 61, Q3: 73) mm/Hg, respectively, and total cholesterol 228 mg/dL (Q1: 202, Q3: 244). Median baseline ultrasensitive estradiol level was 7 pg/mL (Q1: 4, Q3: 15) and hsCRP was 2.45 mg/L (Q1: 1.14, Q3: 6.07). Prior to AI therapy, EndoPAT ratio was 2.18 (Q1: 1.19, Q3: 2.43). Median baseline small arterial elasticity and large arterial elasticity was 4.1 mL/mmHg x100 (Q1: 3.2, Q3: 6.1) and 12.4 mL/mmHg x10 (Q1: 10.4, Q3: 14.2), respectively.

**Table 1:** Cardiovascular characteristics at baseline and 6-month follow-up

	Baseline (all women)	n	Baseline (those with follow-up visit)	n	Follow-up at 6 months	n	Change from baseline	n	p-value
Age	59 (56, 62)	20	58 (56, 60)	14					
Weight (kg)	71 (62, 95)	20	73 (67, 95)	14	73 (65, 101)	13	1 (0, 4)	13	0.052
BMI	26.1 (22.8, 31.4)	20	26.5 (24.4, 31.6)	14	27.1 (23.9, 32.9)	13	0.5 (0.0, 1.3)	13	0.056
Systolic BP	120 (116, 126)	18	120 (115, 124)	13	123 (114, 127)	12	-0.8 (-7.4, 3.6)	12	0.91
Diastolic BP	69 (61, 73)	18	70 (61, 73)	13	69 (62, 71)	12	0.0 (-3.0, 2.6)	12	0.88
Heart rate	65 (61, 75)	18	63 (60, 73)	13	65 (57, 74)	12	0.5 (-6.2, 2.8)	12	0.73
SAE index	4.1 (3.2, 6.1)	18	4.1 (3.2, 6.1)	13	4.3 (4.0, 5.7)	12	0.72 (0.27, 1.08)	12	**0.015**
LAE index	12.2 (10.3, 14.1)	18	12.4 (10.4, 14.2)	13	14.1 (11.0, 17.2)	12	2.10 (-0.70, 3.10)	12	0.13
Fibrinogen	3146 (2737, 3569)	19	3170 (2793, 3666)	14	2933 (2631, 3219)	12	-443 (-672, -68)	12	0.077
hsCRP	2325 (994, 6051)	19	2454 (1137, 6073)	14	2977 (903, 4813)	12	-55 (-830, 951)	12	0.85
Ddimer	7869 (2745, 15,518)	19	8554 (2597, 14,832)	14	5354 (2390, 22,187)	12	70 (-4246, 5116)	12	0.96
tPA	449 (302, 701)	19	452 (299, 712)	14	642 (249, 956)	12	-36 (-66, 137)	12	0.79
vWF	19.8 (9.6, 31.6)	19	19.4 (12.2, 35.2)	14	10.8 (6.4, 17.6)	12	-19.8 (-25.2, -1.8)	12	0.064
PAI1	327 (271, 657)	19	364 (308, 674)	14	499 (194, 816)	12	123 (-49, 249)	12	0.30
Total cholesterol	223 (199, 243)	17	228 (202, 244)	12	213 (210, 229)	11	-1 (-18, 27)	10	0.70
HDL	62 (54, 69)	17	64 (58, 69)	12	73 (61, 77)	11	2 (-3, 14)	10	0.44
LDL	135 (115, 156)	17	143 (121, 159)	12	129 (120, 142)	11	6 (-11, 14)	10	0.65
VLDL	19 (14, 25)	13	17 (14, 22)	10	15 (13, 21)	9	0 (-8, 2)	9	0.48
Triglycerides	102 (70, 126)	17	90 (70, 124)	12	77 (68, 97)	11	2 (-34, 18)	10	0.77
Estradiol	7 (4, 15)	17	7 (4, 15)	12	2 (2, 3)	9	-8 (-12, -3)	8	0.052
EndoPat Ratio	1.88 (0.99, 2.43)	17	2.18 (1.19, 2.43)	13	1.12 (0.85, 1.86)	12	-0.16 (-1.45, -0.02)	12	**0.045**

Table 1: Summaries shown are median (1st quartile, 3rd quartile) and the p-values test for changes between baseline and follow-up. The ‘n’ columns indicate the number of women with non-missing data for each measure. Only 14 of 20 patients with complete follow-up visits were included in study analyses. BMI: Body Mass Index, BP: Blood Pressure, HDL: High-Density lipoprotein, hsCRP: high sensitivity C-Reactive Protein, LAE: Large Arterial Elasticity, LDL: Low-Density Lipoprotein, VLDL: Very-low-Density Lipoprotein, SAE: Small Arterial Elasticity, vWF: Won Willebrand Factor, tPA: Tissue Plasminogen Activator, PAI-1: Plasminogen Activator Inhibitor-1

**Fig. 1:**
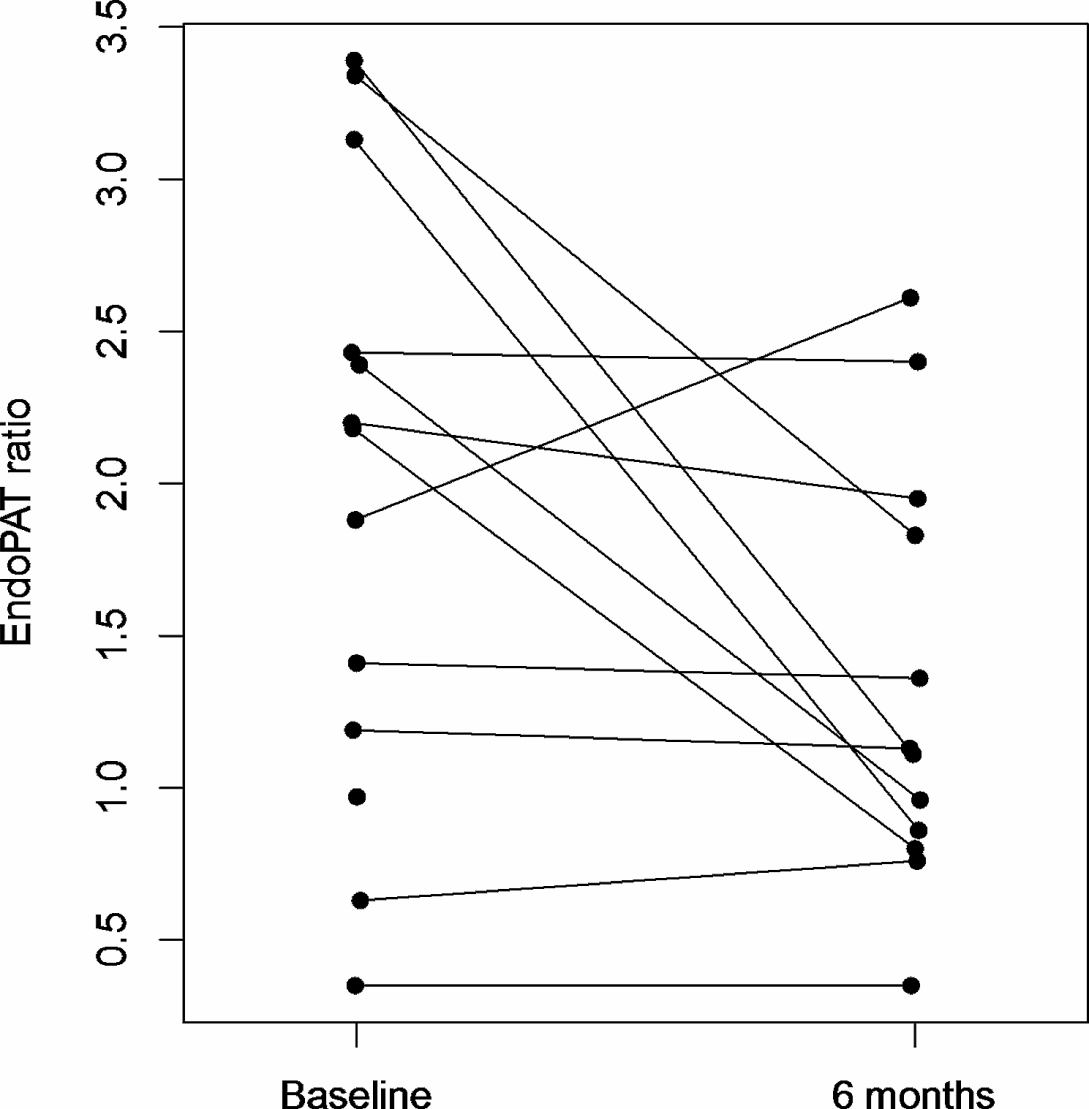
EndoPAT ratio measurements at baseline and 6 months with those from the same woman connected. Figure 1: Of the 14 women, one did not have EndoPAT ratios measured and one was missing the EndoPAT ratio at 6-month follow-up

After six months of AI treatment, EndoPAT® ratio declined to a median 1.12 (Q1: 0.85, Q3: 1.86; *p* = 0.045; Fig. 1) and median estradiol levels decreased to 2 pg/mL (Q1: 2, Q3: 3; *p* = 0.052). There was no evidence of an association between change in EndoPAT® and change in estradiol level (*p* = 0.91). There were no statistically significant changes in small arterial elasticity, which at 6 months was 4.3 mL/mmHg x100 (Q1: 4.0, Q3: 5.7) or large arterial elasticity, which at 6 months was 14.1 mL/mmHg x 10 (Q1: 11.0, Q3: 17.2). There were no statistically significant differences in serum glucose, TC, LDL, HDL, nor hsCRP at 6 months compared to baseline. hsCRP remained elevated at median 2.98 mg/L. Biomarkers of von Willebrand factor, tissue plasminogen activator, and plasminogen activator inhibitor-1 did not change over time.

## Discussion

Endovascular dysfunction is an early sign for atherosclerosis and vascular impairment; therefore, measuring endovascular dysfunction using flow-mediated dilation or EndoPAT helps identify patients who may be at risk for CV events [9, 14]. Our EndoPAT pilot study suggests that postmenopausal breast cancer survivors on AIs therapy develop endothelial dysfunction, a predictor of adverse CV disease. These changes develop while on AIs, correlating with a decline in estradiol levels.

Large clinical studies report higher rates of hypertension, hypercholesterolemia, and ischemic CV disease in postmenopausal breast cancer survivors receiving AIs [7, 8, 15]. The Long Island Breast Cancer study demonstrated increased CV deaths and decreased survival after seven years of treatment with AI [15]; this study, however, did not differentiate whether findings were related to secondary causes such as the development of hypertension, hypercholesterolemia, or directly related to AI therapy. A more recent retrospective cohort of 15,815 breast cancer patients diagnosed 2006–2012 demonstrated an increase in heart failure in those treated over the age of 75 years when treated with an AI compared to tamoxifen. The risk of ischemic heart disease increased in those who took AIs for at least four years (hazard ratio (HR): 2.12; 95% CI: 1.40–3.25) compared to those who took no or had short term exposure to AI [8]. In the study cohort of the UK Clinical Practice Research Datalink of 17,922 breast cancer patients treated with AI, there was an increase in heart failure risk (HR: 1.86; 95% CI: 1.14–3.03) and CV mortality (HR: 1.50; 95% CI: 1.11–2.04) in those treated with AI compared to tamoxifen [7]. Contrarily, a randomized double blinded study compared anastrozole to placebo did not show an increase risk for CV events [16].

CV disease development is multifactorial due to risk factors such as aging, hypertension, hyperlipidemia and tobacco use. This development often begins with endothelial dysfunction and which ultimately leads to atherosclerosis and ischemic events. Inflammation, fibrosis, and estrogen depletion can lead to changes in the endothelium [6, 17, 18]. In this study, treatment of postmenopausal breast cancer with AI was associated with increased endothelial dysfunction, which coincided within six months of starting the medication and declines in estrogen. This finding is significantly lower than the 1.67 EndoPAT level which was previously linked to higher CV events by Shechter et al. [19]. Our prior work also demonstrated higher rates of impaired endothelial function compared with healthy postmenopausal controls [14]. There was a suggestion endothelial changes were associated with a decline in estrogen levels; however, this did not meet statistical significance [14]. Markers of inflammation (hsCRP) remained persistent; other biomarker work did not suggest the etiology of these changes [14].

Given the high prevalence of breast cancer and recommendations for extended use of AIs in postmenopausal women, it is important to investigate further the risk of CV disease development due to AI use as prior studies have been inconclusive. Additionally, with further reductions in estrogen in premenopausal women, where ovarian suppression plus AI is often recommended, understanding the long-term implications of this treatment regimen on overall cardiac health is imperative. In the current literature, few studies have shown the correlation between estrogen levels and endothelial dysfunction. Luca et al. reported study results of ten premenopausal women who showed that estradiol serum levels were inversely proportional to endothelial dysfunction and subsequent CV events by measuring flow-mediated dilation [16]. Given our work, and prior published work, we hypothesize that long-term use of AI can lead to persistent endothelial dysfunction, and further investigation is necessary. In our study, patients were on AI for approximately 5–10 years. As a result, we do not have data on whether these changes, such as EndoPAT® ratio and the elasticity of small and large arterial, are reversible with discontinuation of AI.

## Conclusion and limitations

This study has a few limitations. It is a pilot study, and as such, has a small sample size. Additionally, the population was predominantly Caucasian, limiting the generalizability. This study was designed to detect AI-specific risk because subjects with a known major risk factors such as tobacco use, hypertension, or hyperlipidemia were excluded. These findings set the stage for a larger study to more conclusively determine the association between AI exposure and cardiovascular outcomes. Further studies should evaluate for multivariate associations with modifiable risk factors for CV disease.

Postmenopausal breast cancer survivors prescribed AIs develop endothelial dysfunction, a predictor of adverse CV disease. Understanding the exact mechanisms through further detailed biomarker research long-term, along with potential interventions, such as modifications of CV risk factors, to mitigate these effects is necessary and warrants further investigation.

## Data Availability

The datasets used and/or analyzed during the current study are available from the corresponding author on request.

## Abbreviations

AI: Aromatase inhibitors
ER +: Estrogen-receptor positive
CV: Cardiovascular
CRP: C-reactive protein
HDL: High-density lipoprotein cholesterol
HR: Hazard Ratio
LDL: Low-density lipoprotein cholesterol
TC: Total cholesterol
TG: Triglycerides
tPA: Tissue-type plasminogen activator
PA-1: Plasminogen-activator 1
PAT: Peripheral arterial tonometry
PWA: Pulse wave amplitude
tPA: Tissue-type plasminogen activator
Q: Quartile
UMN: University of Minnesota
